# Differences in Opioid-Related Deaths in the Appalachian Region in 2018-2021 by State and Rural-Urban County Classification

**DOI:** 10.7759/cureus.40480

**Published:** 2023-06-15

**Authors:** Santiago Rengifo, Alice Wu, Patrick Ioffreda, Asif M Ilyas

**Affiliations:** 1 Foundation for Opioid Research & Education, Rothman Orthopaedic Institute, Philadelphia, USA; 2 Orthopedic Surgery, Drexel University College of Medicine, Philadelphia, USA; 3 Orthopedic Surgery, Rothman Orthopaedic Institute at Thomas Jefferson University, Philadelphia, USA; 4 Orthopedic Surgery, Sidney Kimmel Medical College at Thomas Jefferson University, Philadelphia, USA; 5 Foundation for Opioid Research and Education, Rothman Orthopaedic Institute, Philadelphia, USA; 6 Orthopedic Surgery, Sidney Kimmel Medical College at Thomas Jefferson, Philadelphia, USA

**Keywords:** public health, substance use, appalachia, overdose deaths, opioids

## Abstract

Introduction

The rapid increase in opioid-related deaths since the early 2000s is a major US public health concern. This crisis has transitioned from pharmaceuticals to illicit synthetic opioids and street mixtures. This epidemic has significantly impacted the Appalachian region. This study investigated opioid-related death rates among the Appalachian states, focusing on death rates among urban, suburban, and rural counties.

Methods

Opioid-related death data from 2018-2021 for the 13 states that make up the Appalachian region were collected using the Centers for Disease Control and Prevention Wide-ranging Online Data for Epidemiologic Research (CDC WONDER) database. Opioid analgesic overdose deaths were defined using ICD-10 codes X40-X44, X60-X64, and Y10-Y14, where an opioid analgesic was also coded (T40.2-T40.4). US census data was used to calculate opioid-related death rates by population. Counties were classified as urban, suburban, and rural using the 2013 Rural-Urban Continuum Codes from the US Department of Agriculture. The data were descriptively broken down and reported as either percentages or means.

Results

Of the opioid-related deaths between 2018 and 2021, 498 counties were identified in the 13 Appalachian states as having reported at least 10 opioid-related deaths per year. Among these counties, 337 (67.7%) were classified as urban/metropolitan, 138 (27.7%) as suburban, and 23 (4.62%) as rural. Overall, mean opioid-related deaths by populations per 1000 among all counties were 0.24 in 2018, 0.24 in 2019, 0.33 in 2020, and 0.38 in 2021. For urban/metropolitan counties, opioid-related deaths per 1000 gradually increased from 0.23 in 2018 to 0.35 in 2021. For suburban counties, the mean opioid-related deaths per 1000 increased from 0.25 in 2018 to 0.43 in 2021. For rural counties, the mean opioid-related deaths per 1000 increased from 0.43 in 2018 to 0.62 in 2021.

Conclusion

Opioid-related deaths, on average and by population, have risen steadily in the Appalachian region from 2018-2021 across all geographic areas (urban/metropolitan, suburban, rural). Rural counties consistently showed the highest opioid-related deaths per population compared to urban/metropolitan and suburban areas. Addressing social determinants of health such as income level, education level, healthcare access, and community-based interventions is crucial in combating this issue. Community and health system interventions must be implemented to combat the disproportionately high rate of opioid prescribing in the Appalachian region.

## Introduction

The opioid epidemic has become one of the most extensive public health crises, with opioid overdose deaths now ranked as a leading cause of injury-related death in the United States [1]. All populations have experienced increased opioid-related deaths, regardless of race, ethnicity, or geography. However, the magnitude of the impact varies for different populations [2-4]. The heterogeneity in overdose death rates highlights the diverse risk factors driving the crisis and offers insight into its causes.

Overdose deaths caused by opioids occurred in three distinct waves. The "first wave", which began in the 1990s, was driven by aggressive overprescribing of prescription opioids such as hydrocodone and oxycodone and largely affected non-Hispanic White people in small to medium metropolitan areas and non-metropolitan areas. The "second wave" of the opioid crisis began in the late 2000s and was marked by increases in both prescription opioid and heroin-related overdose deaths that affected all populations [2,5,6]. This is primarily seen as a result of prescription opioid restrictions leading many individuals to switch to cheaper and more potent heroin [7,8]. Around 2013, the "third wave" of the opioid epidemic emerged. This was caused by the introduction of synthetic opioids, particularly illicitly manufactured fentanyl (IMF), into the illicit drug supply and profoundly increased overdose deaths among all racial/ethnic groups and geographic regions [9,10]. By 2018, synthetic opioids accounted for nearly 70% of overdose deaths, surpassing heroin as the leading cause of death due to opioids [11]. A recent report noted that fatal overdoses increasingly comprise multiple classes of drugs, with stimulant involvement as a growing concern; however, IMF remained the most frequently occurring substance in toxicology reports [12].

One region that has been hit particularly hard by the opioid epidemic is the Appalachian region. The Appalachian Region spans from northern Mississippi to southern New York and spans 13 states [13]. This region is characterized by persistent Medicaid non-expansion, higher poverty rates, and healthcare access challenges, which are thought to contribute to the higher rates of overdose deaths [14]. Additionally, opioid mortality prevention strategies such as access to naloxone and fentanyl test strips have been blocked or only recently implemented [15,16]. Though reports exist on the effects of this new wave of the opioid epidemic throughout the United States and for some individual states, recent reports on opioid deaths within this region collectively have yet to be seen. This study investigated opioid-related death rates among the Appalachian states, focusing on death rates among urban, suburban, and rural counties.

## Materials and methods

The mortality data were accessed through the Provisional Mortality Statistics Files available through the Centers for Disease Control and Prevention Wide-ranging Online Data for Epidemiologic Research (CDC WONDER) database. De-identified crude rate mortality data were collected on the year, state, and county of occurrence [17]. Drug overdose deaths were identified using the International Classification of Diseases, Tenth Revision (ICD-10) codes based on the underlying cause of death codes X40-44 (unintentional), X60-64 (suicide), and Y10-14 (undetermined intent). Deaths with opioid involvement were identified using the following ICD-10 diagnoses codes: T40.0 (opium), T40.1 (heroin), T40.2 (natural and semisynthetic opioids), T40.3 (methadone), and T40.4 (synthetic opioids other than methadone).

Opioid-related death data were collected from 2018 to 2021 for the 13 states that make up the Appalachian region. These states include Alabama, Georgia, Kentucky, Maryland, Mississippi, New York, North Carolina, Ohio, Pennsylvania, South Carolina, Tennessee, Virginia, and West Virginia. US census population estimates for the respective state and county for the chosen year were used to calculate opioid-related death rates by population [18].

County urban, suburban, and rural status was based on the 2013 Rural-Urban Continuum Codes from the US Department of Agriculture [19], whose classification scheme is organized into nine levels: three urban/metropolitan (metropolitan areas of 1 million population or more, metropolitan areas of 250,000-1 million people, and metropolitan areas of fewer than 250,0000 people), three suburban (urban populations of 20,000 or more and adjacent to a metro area, urban populations of 20,000 or more and not adjacent to a metro area, and urban populations of 2,500 to 19,999 and adjacent to a metro area), and three rural (urban populations of 2,500 to 19,999 and not adjacent to a metropolitan area, completely rural or less than 2,500 urban population yet adjacent to a metro area, and completely rural or less than 2,500 urban population and not adjacent to a metropolitan area). This study went in accordance with these classifications. The data were descriptively broken down and reported as either percentages or means. This study was determined to be exempt from IRB approval.

## Results

**Figure 1:**
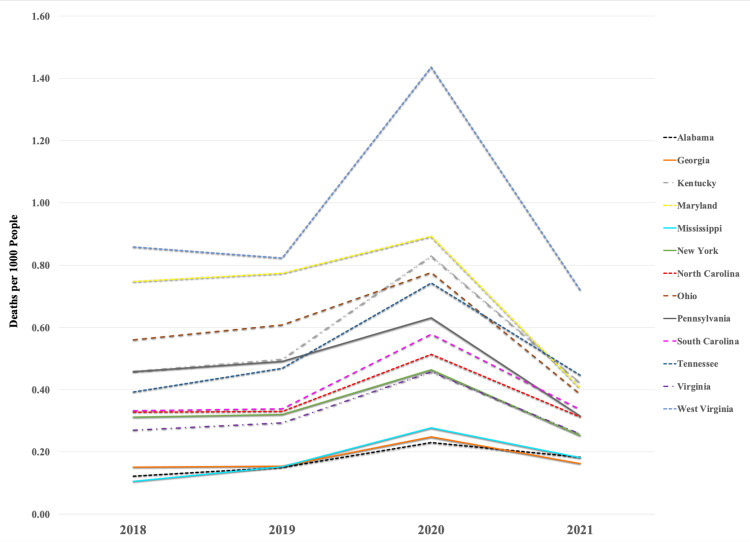
State-by-state change in opioid-related deaths in the Appalachian Region, 2018-2021.

**Figure 2:**
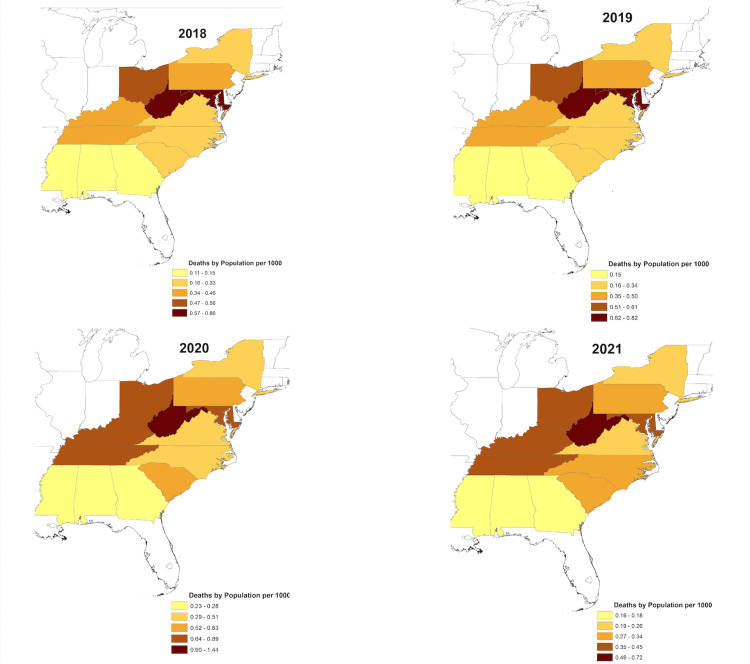
Relative change in opioid-related deaths in the Appalachian Region by state, 2018-2021 This image was created by the authors

A total of 13 states were examined for opioid-related deaths between 2018 and 2021. The total number of opioid-related deaths in these states was 39,128 in 2018, 41,729 in 2019, 60,561 in 2020, and 32,866 in 2021. When calculating the deaths by population for each state, the average number of deaths per 1000 people was 0.39 deaths in 2018, 0.42 in 2019, 0.62 in 2020, and 0.34 in 2021. West Virginia had the highest number of deaths per 1000 people for all years collected, ranging from 0.72-1.44 (Figure 1, 2). Maryland had the second-highest for the years 2018-2020. Alabama had the lowest deaths per 1000 people in 2018 and 2021 (0.10 and 0.181, respectively), while Mississippi had the lowest deaths by population in 2019 and 2020 (0.14 and 0.22, respectively) (Figure 1, 2).

Of the opioid-related deaths between 2018 and 2021, 498 counties from the 13 states that make up the Appalachian region were identified as having reported at least ten opioid-related deaths per year. Of the included 498 counties, 277 (55.6%) counties reported opioid deaths in 2018, 292 (58.6%) in 2019, 394 (79.1%) in 2020, and 488 (97.9%) in 2021. Of the included 498 counties, 337 (67.7%) were classified as urban/metropolitan, 138 (27.7%) as suburban, and 23 (4.62%) as rural. Urban/metropolitan counties included were further classified as 142 (28.5%) in metropolitan areas of 1 million population or more, 121 (24.3%) in metro areas of 250.000-1 million people, and 74 (14.9%) in counties in metro areas of fewer than 250,0000 persons. Suburban counties were subclassified as 72 (14.5%) with urban populations of 20,000 or more and adjacent to a metro area, 11 (2.2%) with urban populations of 20,000 or more, not adjacent to a metro area, and 55 (11.0%) with urban populations of 2,500 to 19,999, adjacent to a metro area. Rural counties were subclassified as 16 (3.2%) with urban populations of 2,500 to 19,999, not adjacent to a metro area, four (0.8%) completely rural or less than 2,500 urban population, adjacent to a metro area, and three (0.6%) completely rural or less than 2,500 urban population, not adjacent to a metropolitan area (Table 1).

**Table 1:** Appalachian counties with opioid-related deaths by RUCC 2013 RUCC - Rural-Urban Continuum Codes as defined by the US Department of Agriculture [19]

Overall counties, N=498		
RUCC	N (% of overall counties)	Description
Urban/metropolitan counties		
1	142 (28.5)	Counties in metro areas of 1 million population or more
2	121 (24.3)	Counties in metro areas of 250,000 to 1 million population
3	74 (14.9)	Counties in metro areas of fewer than 250,000 population
Suburban counties		
4	72 (14.5)	Urban population of 20,000 or more, adjacent to a metro area
5	11 (2.2)	Urban population of 20,000 or more, not adjacent to a metro area
6	55 (11.0)	Urban population of 2,500 to 19,999, adjacent to a metro area
Rural counties		
7	16 (3.2)	Urban population of 2,500 to 19,999, not adjacent to a metro area
8	4 (0.8)	Completely rural or less than 2,500 urban population, adjacent to a metro area
9	3 (0.6)	Completely rural or less than 2,500 urban population, not adjacent to a metro area

The average number of deaths per county in those that had recorded opioid-related deaths in the Appalachian region was 183 (752) in 2018, 193 (790) in 2019, 206 (950) in 2020, and 132 (470) in 2021. For urban/metropolitan counties, the mean was 58.1 (87.6) in 2018, 67.0 (173) in 2019, 73.5 (117) in 2020, and 84.2 (136) in 2021. For suburban counties, the mean was 16 (6.96) in 2018, 17.9 (11.1) in 2019, 20.6 (13.9) in 2020, and 22.9 (15.3) in 2021. For rural counties, the mean was 11 (0.0) in 2018, 14.4 (6.1) in 2020, and 16.8 (7.48) in 2021 (Table 2).

**Table 2:** Annual opioid-related deaths in Appalachian counties by geographic area

	Overall N=498, mean (SD)	Urban/metropolitan N=337, mean (SD)	Suburban N=138, mean (SD)	Rural N=23, mean (SD)
Deaths in 2018	183 (752)	58.1 (87.6)	16.0 (6.96)	11.0 (0.00)
Deaths by population per 1000 in 2018	0.24 (0.40)	0.23 (0.45)	0.25 (0.11)	0.43 (0.22)
Deaths in 2019	193 (790)	67.0 (173)	17.9 (11.1)	NA
Deaths by population per 1000 in 2019	0.24 (0.31)	0.23 (0.34)	0.26 (0.16)	NA
Deaths in 2020	206 (950)	73.5 (117)	20.6 (13.9)	14.4 (6.10)
Deaths by population per 1000 in 2020	0.33 (0.25)	0.29 (0.23)	0.38 (0.24)	0.58 (0.29)
Deaths in 2021	132 (470)	84.2 (136)	22.9 (15.3)	16.8 (7.48)
Deaths by population per 1000 in 2021	0.38 (0.26)	0.35 (0.24)	0.43 (0.27)	0.62 (0.37)

Overall opioid-related deaths by populations per 1000 were 0.24 (0.40) in 2018, 0.24 (0.31) in 2019, 0.33 (0.25) in 2020, and 0.38 (0.26) in 2021. For urban/metropolitan counties, the opioid-related deaths per 1000 were 0.23 (0.45) in 2018, 0.23 (0.34) in 2019, 0.29 (0.23) in 2020, and 0.35 (0.24) in 2021. For suburban counties, the opioid-related deaths per 1000 were 0.25 (0.11) in 2018, 0.26 (0.16) in 2019, 0.38 (0.24) in 2020, and 0.43 (0.27) in 2021. For rural counties, the opioid-related deaths per 1000 were 0.43 (0.22) in 2018, 0.58 (0.29) in 2020, and 0.62 (0.37) in 2021 (Table 2).

## Discussion

The opioid epidemic has significantly impacted the Appalachian region. The current study illustrates how total opioid-related deaths for each Appalachian state, as an average and by population, gradually increased from 2018-2020 and peaked in 2020 before decreasing in 2021. These results are consistent with the national trend of increasing opioid-related deaths since 2013 from the rise of synthetic opioids in the drug market [20,21]. Additionally, the considerable rise in opioid deaths in 2020 may be linked to the COVID-19 pandemic [22,23]. COVID-19-related factors believed to have contributed to the increase in opioid overdose deaths include changes in drug use patterns due to social distancing, limited access to healthcare and community resources, disrupted drug supply chains leading to unfamiliar drug sources, and exacerbated determinants of opioid use such as anxiety, health disparities, and socioeconomic uncertainties [22]. Nevertheless, COVID-19-related trends are difficult to separate from fentanyl and other synthetic opioids that continue to drive fatal overdoses.

The current study illustrates how opioid-related deaths in the Appalachian region as an average and by population have steadily increased from 2018-2021 across all counties by geographic area (i.e., urban/metropolitan, suburban, rural). Most counties included in this study with opioid-related deaths in the Appalachian region from 2018-2021 are categorized as urban/metropolitan counties, with less than a third classified as suburban and <5% rural. One study analyzing the differences between urban and rural counties demonstrated a trend toward prescription opioid misuse among remote, less populated communities. The people most impacted were often older, mostly white, and part of former farm and factory communities. Communities most affected by heroin, synthetic opioids, and synthetic-prescription mixes tended to be more populated, connected to interstates, and with a more ethnically diverse population [24].

The Appalachian region is characterized by persistent Medicaid non-expansion, higher poverty rates, and healthcare access challenges, and all are thought to contribute to the higher rates of overdose deaths [25]. The current study shows rural counties consistently demonstrated the highest rates of opioid-related deaths by population compared to urban/metropolitan and suburban areas. These data align with a recent study that found U.S. drug mortality rates are higher among populations with more blue-collar and service employment, areas with higher opioid prescribing rates, and among more economically disadvantaged counties [26]. These characteristics are reflected most significantly in rural Appalachian counties.

Many social determinants of health contribute to the disparity in opioid-related deaths between Appalachian states and the rest of the country. Prescription Drug Monitoring Programs (PDMPs) show that prescribers in nine of the thirteen Appalachian states prescribe opioids more than the national average [27]. Additionally, many states within the Appalachian region did not legalize the use of fentanyl test strips until 2022, including Alabama, Georgia, West Virginia, Pennsylvania, and Tennessee, despite a correlation between fentanyl test strips and risk reduction behaviors [16,28,29]. Therefore, community and health system interventions must be implemented and improved to combat the disproportionately high rate of opioid prescribing in the Appalachian region.

Addressing discrepancies in social determinants of health such as income level, education level, healthcare access, and community-based interventions will play an essential role in combating opioid-related deaths. As the opioid epidemic rages on and mortality rates increase in the Appalachian region, these states and the counties within them must address the specific community and health system factors contributing to their overdose rates and put in place prevention and treatment strategies that target those most vulnerable in their region.

This study has limitations. To protect patient privacy, the CDC WONDER database excludes counties with fewer than 10 deaths per year, resulting in several unaccounted counties, which may have impacted urban-rural comparisons. Additionally, no information was available on whether other drugs were associated with opioid-overdose deaths. Given that the concurrent use of opioids with certain substances, such as benzodiazepines, may increase the likelihood of overdose [30], the CDC WONDER database would benefit from including this information for use in future studies. For this study, we did not break down the reports to identify the class of opioids associated with overdose deaths. While the CDC WONDER database includes some ICD-10 codes specifying certain classes of opioids in their associated with overdose deaths, such as heroin vs. methadone, as of now, all synthetic opioids are still grouped under one ICD-10 code. As more synthetic opioids continue to be introduced into the drug supply, the database would benefit from expanding the code for synthetic opioids into additional ones identifying the specific opioid involved in the overdose death. Future studies may seek to reference CDC WONDER data with records from each state's department of health websites to collect information on the counties with fewer than 10 opioid-related deaths and gather more information on the specific substances involved in opioid overdose deaths per county.

## Conclusions

This study shows that opioid-related deaths, on average and by population, have risen steadily in the Appalachian region from 2018-2021 across all geographic areas (urban/metropolitan, suburban, rural). Rural counties consistently showed the highest opioid-related deaths per population compared to urban/metropolitan and suburban areas. Addressing social determinants of health such as income level, education level, healthcare access, and community-based interventions is crucial in combating this issue. Community and health system interventions must be implemented to combat the disproportionately high rate of opioid prescribing in the Appalachian region.
